# Cost-effectiveness of Low-Dose Computed Tomography With a Plasma-Based Biomarker for Lung Cancer Screening in China

**DOI:** 10.1001/jamanetworkopen.2022.13634

**Published:** 2022-05-24

**Authors:** Zixuan Zhao, Youqing Wang, Weijia Wu, Yi Yang, Lingbin Du, Hengjin Dong

**Affiliations:** 1 Center for Health Policy Studies, School of Public Health, School of Medicine, Zhejiang University, Hangzhou, China; 2 Department of Cancer Prevention, Cancer Hospital of the University of the Chinese Academy of Sciences/Zhejiang Cancer Hospital, Hangzhou, China; 3 The Fourth Affiliated Hospital, Zhejiang University School of Medicine, Zhejiang, China

## Abstract

**Importance:**

China, which has one-third of the worldwide smoking population, has a substantial cancer burden, with lung cancer being the leading cause of cancer-related death. The effectiveness of lung cancer screening for mortality reduction has been confirmed, but the cost-effectiveness of diverse screening modalities remains unclear.

**Objective:**

To compare the cost-effectiveness of low-dose computed tomography (LDCT) with a biomarker (micro-RNA signature classifier [MSC]) with that of LDCT alone by screening interval and cumulative smoking exposure.

**Design, Setting, and Participants:**

In this economic evaluation, a comparative cost-effectiveness analysis used Markov state transition models that simulated the 1947 to 1971 China birth cohort. Simulated individuals in 8 cohorts of 10 000 entered the study between ages 50 and 74 years and were followed up until death or age 79 years, corresponding to a study period from January 1, 2021, to December 31, 2050. The model was run with a cycle length of 1 year. All the transition probabilities were validated, and health utility values were extracted from published literature. Cost parameters were derived from the databases of local medical insurance bureaus.

**Main Outcomes and Measures:**

Primary outcomes included life-years, quality-adjusted life-years (QALYs), and incremental cost-effectiveness ratios (ICERs) with future costs and outcomes discounted by 5%. Screening strategies with a mean ICER less than Chinese yuan (CNY) 212 676 per QALY gained were deemed to be cost-effective. The cost-effectiveness of 7 alternative screening strategies with a screening starting age of 50 years, minimum cumulative smoking exposure of 20 vs 30 pack-years, and screening interval of annual vs 1 time was estimated, including the 2021 China guideline-recommended strategy (LDCT, annual, 30 pack-years) and the 2018 China guideline-recommended strategy (LDCT, annual, 20 pack-years).

**Results:**

In a simulated population of 80 000 individuals, the conjunctive LDCT and MSC screening strategy was estimated to obtain an ICER of CNY −793 995.17 to 254 417.46 (minimum cumulative smoking exposure, 20-30 pack-years) per QALY gained compared with LDCT screening alone. China’s 2021 guideline-recommended strategy was not cost-effective compared with the 2018 guideline-recommended strategy, with higher costs and fewer QALYs gained; the QALY loss ranged from 0.02 to 0.15 per person and the increase in cost ranged from CNY 945.89 to CNY 5131.29 per person. LDCT and MSC screening beginning at age 70 to 74 years in individuals with a 20 pack-year smoking history was the most cost-effective strategy, with an ICER of CNY −793 995.17 per QALY gained. Lowering the minimum cumulative smoking exposure for screening from 30 to 20 pack-years and maintaining annual screening were associated with greater cost savings regardless of the screening tool.

**Conclusions and Relevance:**

This economic evaluation found that China’s 2018 recommendation for lung cancer screening was more cost-effective than the 2021 recommendation. Moreover, the cost-effectiveness of lung cancer screening was improved when MSC was included with LDCT. These findings may be useful for the modification of guidelines for lung cancer screening.

## Key Points

**Question:**

Is a lung cancer screening strategy that adds a plasma-based biomarker (micro-RNA signature classifier [MSC]) to low-dose computed tomography (LDCT) cost-effective compared with LDCT alone?

**Findings:**

In this model-based economic evaluation of a simulated Chinese population of 80 000 people aged 50 years or older with a history of smoking, conjunctive LDCT and MSC lung cancer screening strategies were estimated to be cost-effective compared with strategies using LDCT alone, with greater incremental cost-effectiveness ratios per quality-adjusted life-year gained.

**Meaning:**

The findings suggest that a conjunctive screening strategy of LDCT and MSC may be more cost-effective than LDCT alone for detecting lung cancer.

## Teaser

This economic evaluation compares the cost-effectiveness of lung cancer screening strategies using low-dose computed tomography (LDCT) and a plasma-based biomarker or LDCT alone in a simulated population of Chinese adults with a history of smoking.

## Introduction

With the increase in the aging population, cancer incidence has markedly increased worldwide. China has a substantial cancer burden, and lung cancer remains the leading cause of cancer-related death, with an estimated 714 699 new deaths in China in 2020.^1^ Similar to recommendations released by the US Preventive Services Task Force based on results from the National Lung Screening Trial,^2,3^ China, which has one-third of the smoking population in the world,^4^ initiated lung cancer screening programs in 2009 and drafted several versions of guidelines for lung cancer screening using low-dose computed tomography (LDCT).^5,6,7^ In 2021, an advisory group on the formulation of guidelines for lung cancer screening, early diagnosis, and early treatment in China updated the 2018 version^6^ of the guideline by increasing the minimum smoking exposure criterion from 20 to 30 pack-years while maintaining the annual screening frequency, the screening start age of 50 years, and the stop age of 74 years.^7^ The modification of the minimum smoking exposure may substantially increase the detection rate of lung cancer among high-risk individuals who meet the inclusion and exclusion criteria. Because the effectiveness of lung cancer screening for mortality reduction has been confirmed,^2,3^ the challenge for lung cancer screening now seems to be the high false-positive rate of LDCT.^2^ Abnormal chest imaging findings may lead to subsequent diagnosis of invasive cancer and related complications according to the current protocols for lung cancer screening in China. The standard procedure in China for participants in whom small pulmonary nodules are detected is performing serial LDCT tests at intervals of 3 to 6 months.^5,6,7^ However, results from the National Lung Screening Trial^8^ demonstrated that 11.7% and 3.5% of the screening findings at baseline and subsequent LDCT tests, respectively, were indeterminate. Meanwhile, the false-positive rates for baseline screening and subsequent tests were 12.8% and 5.3%, respectively.^8^ The prolonged time of uncertainty about the clinical significance of pulmonary nodules, a high use of harmful diagnostic follow-up, the additional frequency of radiation exposure, and increased patient costs and anxiety may contribute to uncertainty about the cost-effectiveness of lung cancer screening.^9,10^ Thus, there is a need to improve the accuracy of lung cancer screening to avoid unnecessary LDCT, diagnostic tests, and extra radiation exposure and to decrease overall morbidity.

Blood- and serum-based biomarkers are promising adjuncts to LDCT in lung cancer screening.^11,12^ Biomarkers with high specificity for early detection of lung cancer may help alleviate the problem of the high false-positive rate when using LDCT alone in a screening program. Micro-RNA signature classifier (MSC), one of the biomarkers that have entered phase 4 of development, may be useful in conjunction with LDCT for lung cancer detection. Few results have been reported to date using these biomarkers in screening practice; thus, the health outcomes associated with adjunctive strategies with LDCT as well as the cost-effectiveness remain unclear.

In this study, we assessed the cost-effectiveness of LDCT and MSC compared with LDCT alone for lung cancer screening. As part of this analysis, we assessed the influence of lowering the minimum cumulative smoking exposure from 30 pack-years, as recommended by China’s 2021 guideline, to 20 pack-years, as recommended by the 2018 guideline, and compared different screening intervals.

## Methods

In this economic evaluation, we simulated 8 cohorts of 10 000 individuals born from 1947 to 1971 in 5-year intervals, thereby targeting the current Chinese population eligible for lung cancer screening based on age. Simulated individuals entered the study between age 50 and 74 years and were followed up until death or age 79 years (mean life expectancy in China), corresponding to a study period from January 1, 2021, to December 31, 2050. We used a Markov state transition model with this lifetime horizon to simulate the natural history and the screening process for lung cancer. The model was run with a cycle length of 1 year. The study was conducted according to the Consolidated Health Economic Evaluation Reporting Standards (CHEERS) and was approved by the ethics committee of the Taizhou Cancer Hospital; informed consent was not applicable because this was a modeling study.

The target strategies were based on a screening start age of 50 to 74 years. Strategy 0 was the 2021 guideline-recommended strategy of LDCT screening annually with a minimum cumulative smoking exposure of 30 pack-years; strategy 1, LDCT screening once with a minimum cumulative smoking exposure of 30 pack-years; strategy 2 (2018 guideline-recommended strategy), LDCT screening annually with a minimum cumulative smoking exposure of 20 pack-years; strategy 3, LDCT screening once with a minimum cumulative smoking exposure of 20 pack-years; strategy 4, LDCT and MSC screening annually with a minimum cumulative smoking exposure of 30 pack-years; strategy 5, LDCT and MSC screening once and a minimum cumulative smoking exposure of 30 pack-years; strategy 6, LDCT and MSC screening annually with a minimum cumulative smoking exposure of 20 pack-years; and strategy 7, LDCT and MSC screening once with a minimum cumulative smoking exposure of 20 pack-years. Strategies 1 to 7 were compared with strategy 0. Of note, a positive test result for MSC was used as the inclusion criterion for conspicuous nodules detected by LDCT. The scheme diagram is shown in the eFigure in the Supplement. The rationale for the strategy of screening only once was attributable to screening practices under limited financial support for lung cancer screening programs in China at present.^7^ To attempt to reflect this situation, we assessed whether screening only once would be cost-effective if annual screening was not available and under which circumstances screening would be cost-effective. The cost-effectiveness of screening strategies was evaluated from the perspective of the China health care sector, assuming 100% adherence to screening.

### Model Description

**Figure 1.**
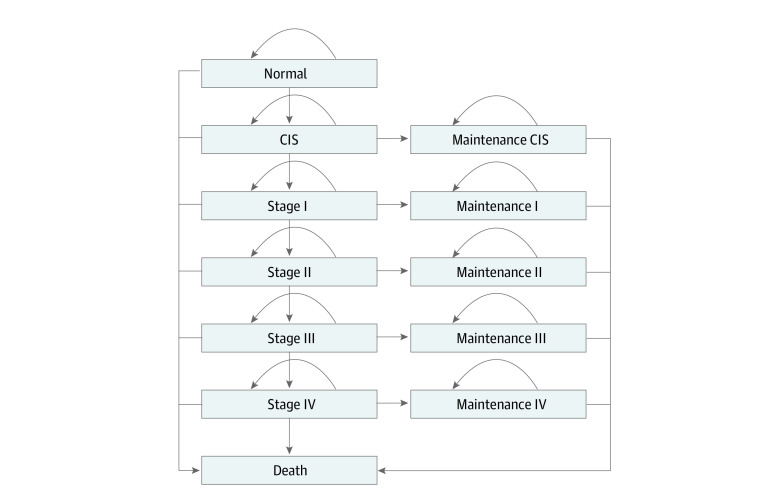
Markov Process Model CIS indicates carcinoma in situ.

The Markov state transition model consisted of 12 health states: normal; carcinoma in situ (CIS); stages I, II, III, and IV; maintenance for each stage of cancerous lesion; and death (Figure 1). All cancerous lesions were classified based on the *American Joint Committee on Cancer Staging Manual, 8th edition*.^13^ Stages I and III could not be divided into stages IA, IB, IIIA, and IIIB because data for these stages were not available for Chinese clinical practice or the extracted cancer registry data.^14^ The maintenance states were defined as periodic follow-up after the main treatment for each stage, and experts’ opinions were adopted for the establishment of these states. The Markov state transition model and all the simulations were created using Treeage Pro, version 2021 (Treeage Software).

### Model Input Parameters

As in many Markov modeling studies, there were 3 components of the input parameters: costs, health utility, and transition probabilities. Costs associated with the screening program consisted of expenses for public advertising, screening invitation management, staff salary, consumable materials, and depreciation of screening machinery and were estimated based on an interview with the work team of the Wenling Lung Cancer Screening Program. Costs associated with diagnostic procedures and treatment were obtained from the internal database of the local medical insurance bureau, which included 4947 patients and 107 248 relevant records. Specifically, the cost of maintenance by clinical stage was 10% of the treatment cost. All the costs in this study are expressed in Chinese yuan (CNY) and were discounted to the 2018 price level at a discount rate of 5%. The life-years of simulated individuals were adjusted for quality of life using published health utility scores by clinical stage.^15,16^ We did not adjust for quality of life for the first-round positive cases (those with positive results on LDCT screening or conjunctive screening but not yet diagnosed by biopsy) because the National Lung Screening Trial reported that indeterminate results did not affect quality of life.^17^ We applied pooled quality-of-life scores taken from a meta-analysis by Sturza^15^ and a survey by Chen.^16^ The initial lung cancer incidence was estimated as a multiplicative function of smoking rate, age, and gender-specific incidence parameters.^18,19,20^ Rates of smokers with minimum cumulative smoking exposure of 20 pack-years and 30 pack-years were applied.^19,20^ The proportion of lung cancer detected by strategies using LDCT was derived from screening results of the Wenling Lung Cancer Screening Program, which was initiated in 2018 to conduct annual LDCT screening for local populations at high risk of lung cancer in Zhejiang with follow-up for 3 years. A total of 10 175 asymptomatic individuals were screened by the program in 2018, and 65 patients were diagnosed with lung cancer; details of the proportions by cancer stage are presented in Table 1. Annual screening followed the same screening protocol as in the Cancer Screening Program in Urban China, which determined cancer by morphologic features and the size of the nodule.^21^ The proportion of lung cancer detected by strategies using LDCT and MSC was derived from results of the Multicentric Italian Lung Detection trial.^27^ The probability of health to all-cause death was estimated as all-cause mortality for smokers by age.^23,24^ The probability of lung cancer–specific death came from a study by Zhang et al^22^ and was also adjusted for smoking status.^25,26^ The probability that a cancerous state progressed to a more advanced state or to a maintenance state is detailed by cancer stage in Table 1 according to previous work.^15,16,18,19,20,21,22,23,24,25,26,28,29,30^ The specificity and sensitivity of LDCT and LDCT with MSC for lung cancer screening were derived from a meta-analysis by Chu et al.^11^

**Table 1.** Input Parameters of Markov Model for Lung Cancer Screening

Variable	Base-case value	Distribution	Source
Lung cancer incidence per 100 000 general population by age group			
50-54 y			He and Chen^18^
Female	89.6626	Beta	
Male	81.0559		
55-59 y			
Female	112.4574	Beta	
Male	162.0833		
60-64 y			
Female	154.6871	Beta	
Male	256.0943		
65-69 y			
Female	190.2521	Beta	
Male	373.6808		
70-74 y			
Female	242.6310	Beta	
Male	498.0681		
RR for >20 pack-years	2.84	Beta	Estimated (Yuan et al^19^)^a^
RR for >30 pack-years	3.87	Beta	Sun et al^20^
Proportion of lung cancer cases detected by LDCT with MSC by stage			
CIS	0	Beta	Estimated (Chen et al^21^)^a^
I	0.5441	Beta	
II	0.0570	Beta	
III	0.1195	Beta	
IV	0.2794	Beta	
Proportion of lung cancer detected by LDCT only by stage			
CIS	0.0370	Beta	Wenling Lung Cancer Screening Program
I	0.6852	Beta	
II	0.0370	Beta	
III	0.1852	Beta	
IV	0.0556	Beta	
LDCT			
Sensitivity, %	79	Beta	Zhang et al^22^
Specificity, %	81	Beta	
LDCT with MSC			
Sensitivity, %	69	Beta	Zhang et al^22^
Specificity, %	96	Beta	
All-cause mortality by age group, %			
50-54 y	0.45	Beta	Estimated (National Bureau of Statistics of China^23^; Liu et al^24^)^a^
55-59 y	0.65	Beta	
60-64 y	1.08	Beta	
65-69 y	1.88	Beta	
70-74 y	3.36	Beta	
75-79 y	5.40	Beta	
Lung cancer deaths per 100 000 general population by age group			
50-54 y	28.81	Beta	Liu et al^24^
55-59 y	52.86	Beta	
60-64 y	101.93	Beta	
65-69 y	153.34	Beta	
70-74 y	248.57	Beta	
Lung cancer stage transition probabilities at 1 y			
CIS to stage I	0.0980	Beta	Chen et al^21^
Stage I to stage II	0.3682	Beta	Sun et al^20^
Stage I to stage III	0.0328	Beta	
Stage I to stage IV	0.0745	Beta	
Stage II to stage III	0.2260	Beta	
Stage II to stage IV	0.1510	Beta	
Stage III to stage IV	0.1455	Beta	
CIS to death	0	Beta	Estimated (Zhang et al^22^; Wang et al^25^; Hong et al^26^)^a^
Stage I to death	0.1739	Beta	
Stage II to death	0.2842	Beta	
Stage III to death	0.4626	Beta	
Stage IV to death	0.5880	Beta	
Utility			
CIS	0.87	Beta	Sturza^15^
Stage I	0.84	Beta	Chen^16^
Stage II	0.84	Beta	
Stage III	0.87	Beta	
Stage IV	0.75	Beta	
Cost, CNY			
Screening with LDCT	245.86	Gamma	Survey data^b^
Screening with MSC	400.00	Gamma	
Prediagnosis	628.36	Gamma	
Biopsy diagnosis	1232.44	Gamma	
Treatment			
CIS	47 341.85	Gamma	
Stage I	53 344.51	Gamma	
Stage II	83 365.95	Gamma	
Stage III	90 643.18	Gamma	
Stage IV	116 471.34	Gamma	

Abbreviations: CIS, carcinoma in situ; CNY, Chinese yuan; LDCT, low-dose computed tomography; MSC, micro-RNA signature classifier; RR, relative risk ratio.

^a^ Values were calculated based on data from the cited sources because they could not be extracted directly from those sources.

^b^ Data were collected as part of this study.

### Outcome Measures

Primary outcomes included life-years, quality-adjusted life-years (QALYs), and incremental cost-effectiveness ratios (ICERs) of different screening strategies compared with the 2021 guideline-recommended strategy. The ICER was calculated by dividing the incremental costs by the incremental QALYs gained for each screening strategy compared with the 2021 guideline-recommended strategy. Alternative strategies that yielded more life-years or QALYs at lower cost compared with the baseline strategy were designated as dominant strategies, and the strategies that yielded more life-years or QALYs at higher cost were designated as extended dominated strategies. In accordance with World Health Organization recommendations, 1 to 3 times the gross domestic product (GDP) per capita in China (CNY 70 892-212 676) per QALY gained was used as the willingness-to-pay threshold to define strongly cost-effective and weakly cost-effective strategies.^31^

### Sensitivity Analysis

Univariate sensitivity analyses were conducted to assess the sensitivity of the results to changes in the value of important model input parameters including screening cost, maintenance cost, discount rate, Consumer Price Index rate, and specificity and sensitivity of LDCT and LDCT with MSC. The cost of screening and the maintenance cost and Consumer Price Index rate were set to vary by 30% compared with base-case values. The discount rate was set to range from 0% to 8%. The sensitivity and specificity were set to range from 0.63 to 0.95 and 0.65 to 0.97, respectively, for LDCT only and from 0.41 to 0.98 and 0.81 to 0.99, respectively, for LDCT with MSC. Input parameters were randomly drawn from beta or gamma distribution (Table 1 and eTable 1 in the Supplement). Probabilistic sensitivity analyses were performed to address the joint uncertainties in the values of input parameters using 10 000 iterations. The probability that each screening strategy was cost-effective at a given willingness-to-pay threshold was calculated by counting the number of times per 10 000 iterations that the ICER was below the specified threshold.

## Results

### Base-Case Analysis

Table 2 provides the results for the simulated population of 80 000 adults aged 50 years or older. The results from our model suggested a gain of 0.02 to 0.15 QALYs per person using the 2018 guideline-recommended strategy (strategy 2) compared with the 2021 guideline-recommended strategy (strategy 0). Meanwhile, the cost savings ranged from CNY 945.89 to CNY 5131.29 per person, indicating that the 2021 guideline-recommended strategy was not cost-effective compared with the 2018 recommendation. Annual screening by LDCT with minimum cumulative smoking exposure of 20 pack-years (strategy 2) and annual screening with LDCT with MSC with minimum cumulative smoking exposure of 20 pack-years (strategy 6) were dominant strategies, with CNY 26 039.00 and CNY 40 517.15 saved per QALY gained, respectively, for individuals starting screening from age 50 to 74 years. Screening once by LDCT with a minimum cumulative smoking exposure of 30 pack-years (strategy 1) and once with LDCT with MSC with a minimum cumulative smoking exposure of 30 pack-years (strategy 5) for individuals starting screening from age 50 to 74 years were the extended dominated strategies. Compared with the 2021 guideline-recommended strategy, the ICER for screening once by LDCT or LDCT with MSC increased from CNY 255 943.68 per QALY gained for individuals with a screening start age of 50 years to CNY 75 360.72 per QALY gained for individuals with a screening start age of 70 years. The conjunctive LDCT and MSC screening strategy was estimated to obtain an ICER of CNY −793 995.17 to 254 417.46 per QALY gained compared with LDCT screening alone.

**Table 2.** Outcomes of the Base-Case Analysis of Alternative Strategies Compared With the 2021 Guideline-Recommended Strategy

Age at screening start, strategy^a^	Cost, CNY, millions	LYs, in 10 000s	QALYs, in 10 000s	ICER
50 y				
0	2850.60	131.68	131.34	NA
1	2178.78	131.24	130.95	170 916.70
2	2466.19	133.09	132.82	–26 039.00^b^
3	2162.75	131.36	131.07	255 943.68
4	2711.3	131.39	131.07	51 816.20
5	2169.29	131.23	130.94	169 106.85
6	2337.47	132.86	132.61	–40 517.15^b^
7	2154.25	131.35	131.07	253 821.97
55 y				
0	2624.19	117.02	116.66	NA
1	2064.38	116.55	116.25	136 073.86
2	2303.03	118.16	117.86	–26 795.85^b^
3	2044.98	116.68	116.38	205 164.59
4	2504.53	116.71	116.37	41 735.56
5	2053.65	116.54	116.23	133 629.86
6	2191.01	117.9	117.62	–44 883.22^b^
7	2035.40	116.67	116.37	201 037.86
60 y				
0	2294.40	100.21	99.85	NA
1	1856.28	99.76	99.45	110 764.01
2	2056.71	100.96	100.65	–29 686.70^b^
3	1830.93	99.89	99.60	184 393.06
4	2199.91	99.90	99.57	33 469.96
5	1844.61	99.73	99.43	107 694.64
6	1966.45	100.69	100.41	–58 563.85^b^
7	1820.56	99.88	99.58	177 400.98
65 y				
0	1865.10	81.30	80.97	NA
1	1554.41	80.92	80.64	93 276.04
2	1709.14	81.66	81.37	–39 002.09^b^
3	1538.04	80.99	80.72	126 336.89
4	1299.61	81.03	80.73	26 858.57
5	1130.39	80.89	80.61	89 011.27
6	1205.02	81.43	81.16	–121 641.99^b^
7	1526.50	80.96	80.69	119 028.39
70 y				
0	1799.12	59.76	59.51	NA
1	1542.15	59.53	59.31	82 105.77
2	1644.69	59.91	59.68	–56 880.33^b^
3	1538.04	59.58	59.36	122 600.49
4	1266.90	59.58	59.35	19 748.11
5	1117.97	59.49	59.27	75 360.72
6	1171.92	59.74	59.53	–793 995.17^b^
7	1100.09	59.36	59.33	108 465.86

Abbreviations: CNY, Chinese yuan; CPI, Consumer Price Index; ICER, incremental cost-effectiveness ratio; LDCT, low-dose computed tomography; LY, life-year; MSC, micro-RNA signature classifier; NA, not applicable; QALY, quality-adjusted life-year.

^a^ Strategy 0 was the 2021 guideline-recommended strategy of LDCT screening annually with a minimum cumulative smoking exposure of 30 pack-years; strategy 1, LDCT screening once with a minimum cumulative smoking exposure of 30 pack-years; strategy 2 (2018 guideline-recommended strategy), LDCT screening annually with a minimum cumulative smoking exposure of 20 pack-years; strategy 3, LDCT screening once with a minimum cumulative smoking exposure of 20 pack-years; strategy 4, LDCT and MSC screening annually with a minimum cumulative smoking exposure of 30 pack-years; strategy 5, LDCT and MSC screening once and a minimum cumulative smoking exposure of 30 pack-years; strategy 6, LDCT and MSC screening annually with a minimum cumulative smoking exposure of 20 pack-years; and strategy 7, LDCT and MSC screening once with a minimum cumulative smoking exposure of 20 pack-years.

^b^ Dominant.

A lung cancer screening program scheduled only once was cost-effective using strategy 1 (LDCT with smoking exposure of 30 pack-years) or 5 (LDCT and MSC with smoking exposure of 30 pack-years) with a screening start age of 50 to 74 years and using strategy 3 (LDCT with smoking exposure of 20 pack-years) or 7 (LDCT and MSC with smoking exposure of 20 pack-years) with a screening start age of 55 to 74 years. Among these, strategy 5 in individuals who started screening at age 50 to 74 years was the most cost-effective, with an ICER ranging from CNY 75 360.72 to CNY 169 106.85 per QALY gained, and strategy 7 was most effective in individuals who began screening at 70 to 74 years, with an ICER of CNY −793 995.17 per QALY gained.

### Sensitivity Analysis

**Figure 2.**
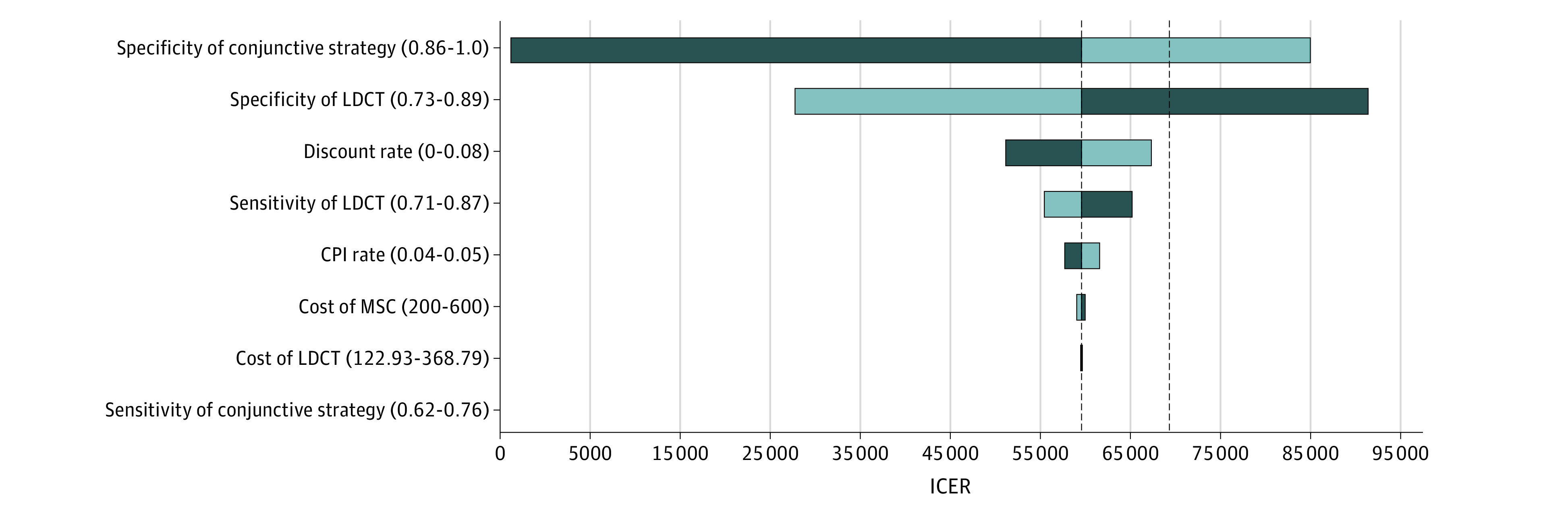
Univariate Sensitivity Analyses of Annual Lung Cancer Screening With Low-Dose Computed Tomography (LDCT) Alone vs LDCT With Micro-RNA Signature Classifier (MSC) The incremental cost-effectiveness ratio (ICER) was defined as the cost of China’s 2018 guideline-recommended lung cancer screening strategy (annual LDCT screening with a minimum cumulative smoking exposure of 20 pack-years) minus the cost of the strategy using annual conjunctive LDCT and MSC screening with a minimum smoking exposure of 20 pack-years divided by the quality-adjusted life-years (QALYs) gained using the 2018 guideline-recommended strategy minus the QALYs gained using the conjunctive strategy when important input parameters were varied for both strategies (1 strategy at a time) by 10% to 30% higher or lower than their base-case values (eTable 1 in the Supplement). The baseline incremental ICER was Chinese yuan (CNY) 61 348.17, and the baseline willingness to pay was CNY 70 692.00. Dark blue represents decreases in input parameters, and light blue, increases in input parameters. The values in parentheses for each parameter indicate the range for that parameter. CPI indicates Consumer Price Index.

In the sensitivity analysis, the results were robust to the changes of the values from the base-case analysis, with no variation exceeding CNY 212 676 per QALY gained (Figure 2). The ICERs of screening strategies were most sensitive to changes in specificity values for LDCT alone and conjunctive screening with LDCT and MSC. The per capita GDP (CNY 70 892) served as the threshold for absolute cost-effectiveness; the efficiency ranges for each parameter included in the sensitivity analysis are provided in Figure 2. When assuming the specificity of LDCT with MSC would be better than 97.5%, the cost-effectiveness of annual screening with LDCT and MSC would be absolutely cost-effective with an ICER less than 1 multiplied by the GDP per capita in China. Annual use of LDCT alone had already been deemed absolutely cost-effective with its baseline specificity value of 81.0% (which was higher than the threshold of 78.4%).

**Figure 3.**
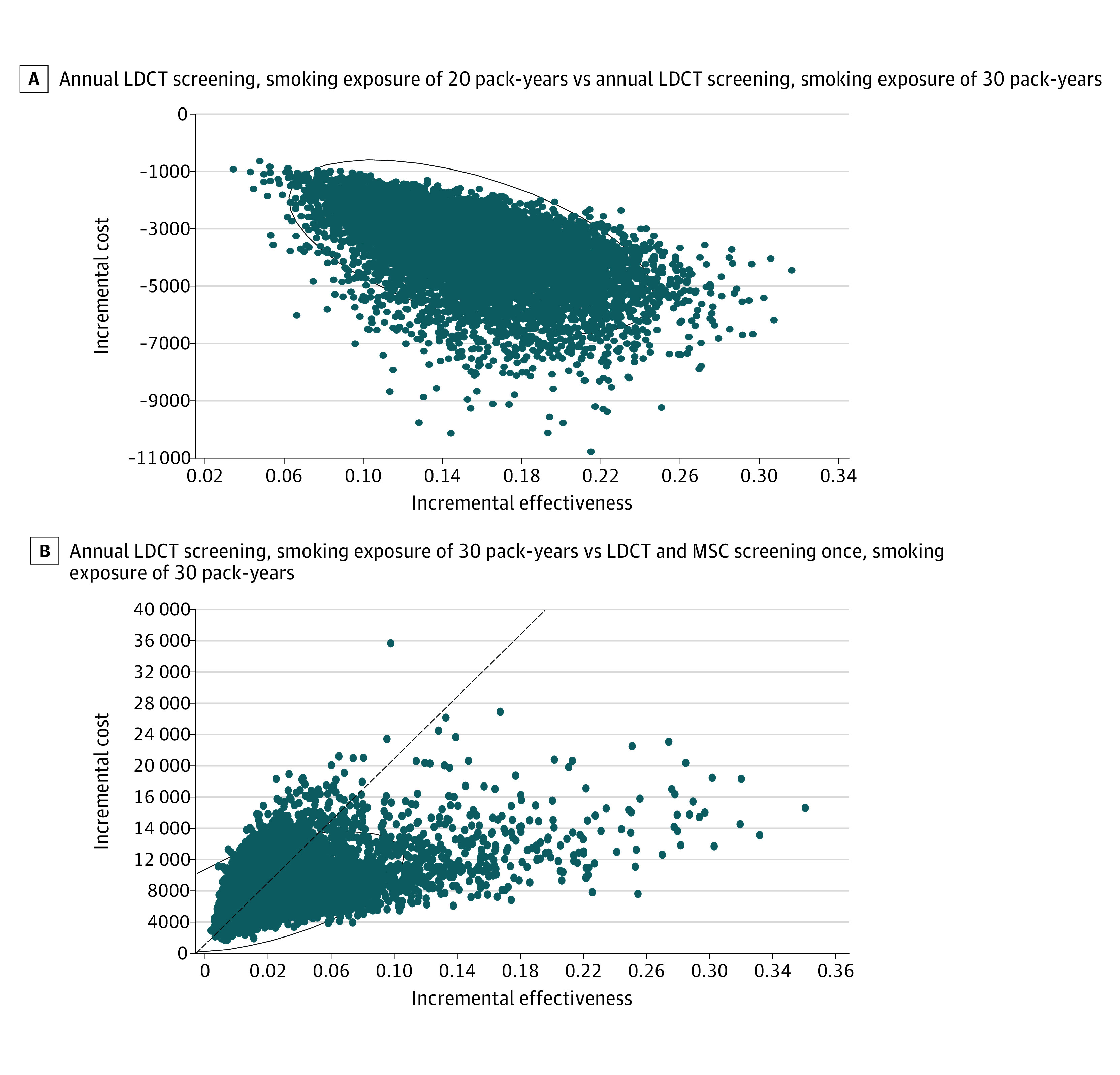
Probabilistic Sensitivity Analyses of Diverse Screening Strategies for Lung Cancer Ovals represent 95% CIs, and dots indicate the results of each iteration in the probabilistic sensitivity analysis. B, The dashed diagonal line indicates the willingness-to-pay threshold of Chinese yuan (CNY) 212 276 per quality-adjusted life-year gained. The dots above the dashed line indicate cost-effectiveness. Incremental costs are in CNY. LDCT indicates low-dose computed tomography; MSC, micro-RNA signature classifier.

The probability sensitivity analysis demonstrated that the results of the base-case analysis were robust to simultaneous changes in important input parameters (Table 1 and eTable 2 in the Supplement). Of note, compared with the 2021 guideline-recommended strategy, the 2018 guideline-recommended strategy had a 100% likelihood of being cost-effective when the willingness-to-pay threshold was CNY 70 892 (GDP per capita in China). However, compared with strategy 5 (LDCT and MSC once with smoking exposure of 30 pack-years), the 2021 guideline-recommended strategy had a 38.12% likelihood of being cost-effective when the willingness-to-pay threshold was CNY 212 676 (3 times the GDP per capita in China) (Figure 3).

## Discussion

To our knowledge, this study is the first to use a comparative modeling approach to assess the cost-effectiveness of China’s 2021 guideline-recommended screening process for lung cancer and a conjunctive strategy using LDCT and a plasma-based biomarker (MSC). When alternative strategies were compared with the 2021 guideline-recommended strategy, the 2018 guideline-recommended strategy and strategy 6 (LDCT and MSC annually with a minimum cumulative smoking exposure of 20 pack-years) were considered dominant strategies, indicating a better economic performance for lung cancer screening among those with smoking exposure of 20 pack-years. Although there is no official recommendation for 1-time screening for lung cancer in China, 1-time LDCT screening was associated with significantly lower lung cancer mortality and all-cause mortality in a large Chinese population^32^; thus, we simulated the strategy using different screening tools and an inclusion criterion of smoking exposure. Using a willingness-to-pay threshold of CNY 212 676 (approximately $37 500 USD and £27 256 GBP in 2021), the screening strategies using 1-time LDCT or LDCT with MSC were cost-effective in individuals with a minimum cumulative smoking exposure of 30 pack-years (strategies 1 and 5). As a reference, the common willingness-to-pay thresholds in the US and UK are $100 000 USD and £20 000 to £30 000 GBP, respectively, per QALY gained.^33,34^ We found that the results of the cost-effectiveness analysis were associated with the specificity of both LDCT and LDCT with MSC more than with any other input parameters. Once the specificity of LDCT was 78.3% or more, the strategy of using LDCT alone was absolutely cost-effective. When using LDCT and MSC, the specificity for the conjunctive strategy needed to increase to 97.5% to make the ICER less than 1 multiplied by the GDP per capita in China. Toumazis et al^33^ found a similar result: the 2021 US Preventive Services Task Force recommendation on lung cancer screening was cost-effective compared with the 2013 recommended screening strategy. In 2021, the US Preventive Services Task Force lowered the starting age for lung cancer screening from 55 to 50 years and the minimum cumulative smoking exposure from 30 to 20 pack-years compared with its 2013 version, whereas the change was the opposite in China. China issued the 2021 guideline recommendation to increase the minimum cumulative smoking exposure from 20 to 30 pack-years and maintain the screening start age of 50 years vs the 2018 recommendation. However, this study’s finding that adopting a minimum cumulative smoking exposure of 20 pack-years for lung cancer screening may be more cost-effective is applicable to both US and Chinese circumstances.

As for alternative strategies using conjunctive screening, a recently published cost-effectiveness study revealed that incorporating a diagnostic biomarker with at least a medium sensitivity profile and 90% specificity and that costs $250 or less was cost-effective, with an ICER less than $100 000 per QALY gained.^35^ However, the existing biomarkers have mainly been tested retrospectively in patients with lung cancer, and their accuracy needs further investigation in prospective studies among asymptomatic individuals.^11,27,36^ This study demonstrated that when using a willingness-to-pay threshold of CNY 212 676, LDCT and MSC screening annually with a smoking exposure of 30 pack-years (strategy 4), LDCT and MSC screening once with a smoking exposure of 30 pack-years (strategy 5), LDCT and MSC screening annually with a smoking exposure of 20 pack-years (strategy 6), and LDCT and MSC screening once with a smoking exposure of 20 pack-years (strategy 7) were cost-effective with the starting age of screening postponed from 50 to 55 years. Once the specificity of LDCT with MSC was better than 97.5%, the cost-effectiveness of annual screening with LDCT and MSC would be absolutely cost-effective. With regard to policy implications, the current lung cancer screening programs in China are sponsored by the central and local government by providing free 1-time LDCT screening for high-risk populations regionally; the plasma-based biomarker (MSC) is not covered by medical insurance solely for an outpatient visit, and the cost of screening for MSC is nearly double the cost of LDCT alone. Although the conjunctive screening strategy was deemed to be cost-effective in this study, the higher out-of-pocket cost was associated with lower use.^37^ There is a need to provide bonuses for participants in the conjunctive screening strategy to ensure uptake of the screening program once MSC screening is included.

### Limitations

This study has limitations. First, the uptake and adherence rates for diagnostic procedures were assumed to be 100%, although in practice, the uptake of lung cancer screening has varied in different studies and has been estimated to be between 34.41% and 48.21% in China.^38,39,40^ The data also were restricted to LDCT screening alone. Thus, we evaluated the screening strategies under the assumption of perfect adherence to capture the full extent of benefits associated with lung cancer screening among different screening strategies. Second, the possibility of increased cancer risk (eg, breast or thyroid cancer) associated with the cumulative radiation burden of LDCT was not considered in our model; however, several studies have demonstrated that the potential benefit of lung cancer screening in preventing death is greater than the potential harm of increased risk of newly developed cancer.^41,42,43,44^ Moreover, we aimed to assess the comparative cost-effectiveness between LDCT screening and LDCT with MSC screening, and individuals in all strategies underwent the same number of LDCT scans so that the cost-effectiveness rankings would not be affected. Third, potential behavior changes (eg, smoking cessation) associated with screening were not considered. The effectiveness of smoking cessation interventions is currently evaluable only by studies conducted in the US, Canada, and other Western countries,^45,46,47^ and smoking cessation may have a diverse effect among Asian populations. However, owing to our cohort-based modeling approach, we were not able to reliably estimate the actual duration of heavy smoking behavior or the time passed since individuals changed their behavior. This may have affected the estimation of the QALYs that each cohort could obtain and led to underestimation of the cost-effectiveness for lung cancer screening.^48^

## Conclusions

This economic evaluation found that the 2018 recommendation for lung cancer screening in China was cost-effective compared with the 2021 recommendation. Moreover, the cost-effectiveness of lung cancer screening was improved when screening tools were expanded to include a plasma-based biomarker (MSC). If 1-time screening is used, LDCT with MSC screening in former smokers with a 30 pack-year smoking history might be the most cost-effective approach in China.

## Notes

Supplement. **eTable 1.** Sensitivity Analysis Range of Input Parameters for the Tornado Diagram **eTable 2.** Distributions for Input Parameters in Probabilistic Sensitivity Analysis **eFigure.** Scheme Diagram

## References

[1] Global Cancer Observatory. China. International Agency for Research on Cancer, World Health Organization; 2020. Accessed November 2021. https://gco.iarc.fr/today/data/factsheets/populations/160-china-fact-sheets.pdf

[2] de Koning HJ, van der Aalst CM, de Jong PA, . Reduced lung-cancer mortality with volume CT screening in a randomized trial. N Engl J Med. 2020;382(6):503-513. doi:10.1056/NEJMoa1911793 31995683

[3] Aberle DR, Adams AM, Berg CD, ; National Lung Screening Trial Research Team. Reduced lung-cancer mortality with low-dose computed tomographic screening. N Engl J Med. 2011;365(5):395-409. doi:10.1056/NEJMoa1102873 21714641 PMC4356534

[4] Bender E. Epidemiology: the dominant malignancy. Nature. 2014;513(7517):S2-S3. doi:10.1038/513S2a 25208070

[5] China Lung Cancer Prevention and Treatment Alliance. Chinese expert consensus on early diagnosis of primary bronchial lung cancer. Article in Chinese. *Chinese J Tuberculosis Respiration*. 2014;37(3):172-176.

[6] Zhou Q, Fan Y, Wang Y, . China National Lung Cancer Screening Guideline with low-dose computed tomography (2018 version). Article in Chinese. Zhongguo Fei Ai Za Zhi. 2018;21(2):67-75. 29526173 10.3779/j.issn.1009-3419.2018.02.01 PMC5973012

[7] He J, Li N, Chen W, . China lung cancer screening and early diagnosis and early treatment guidelines (2021, Beijing). Article in Chinese. *Zhonghua Zhong Liu Za Zhi*. 2021;43(3):243-268. doi:10.3760/cma.j.cn112152-20210119-00060 33752304

[8] Pinsky PF, Gierada DS, Black W, . Performance of Lung-RADS in the National Lung Screening Trial: a retrospective assessment. Ann Intern Med. 2015;162(7):485-491. doi:10.7326/M14-2086 25664444 PMC4705835

[9] Sozzi G, Boeri M. Potential biomarkers for lung cancer screening. Transl Lung Cancer Res. 2014;3(3):139-148. 25806293 10.3978/j.issn.2218-6751.2014.06.04 PMC4367694

[10] Goulart BH, Ramsey SD. Moving beyond the national lung screening trial: discussing strategies for implementation of lung cancer screening programs. Oncologist. 2013;18(8):941-946. doi:10.1634/theoncologist.2013-0007 23873718 PMC3755932

[11] Chu GCW, Lazare K, Sullivan F. Serum and blood based biomarkers for lung cancer screening: a systematic review. BMC Cancer. 2018;18(1):181. doi:10.1186/s12885-018-4024-3 29439651 PMC5812229

[12] Borg M, Wen SWC, Nederby L, . Performance of the EarlyCDT® Lung Test in detection of lung cancer and pulmonary metastases in a high-risk cohort. Lung Cancer. 2021;158:85-90. doi:10.1016/j.lungcan.2021.06.010 34130044

[13] Amin MB, Edge S, Greene F, , eds; American Joint Committee on Cancer. AJCC Cancer Staging Manual. 8th ed. Springer-Verlag; 2017. doi:10.1007/978-3-319-40618-3

[14] Wei W, Zeng H, Zheng R, . Cancer registration in China and its role in cancer prevention and control. Lancet Oncol. 2020;21(7):e342-e349. doi:10.1016/S1470-2045(20)30073-5 32615118

[15] Sturza J. A review and meta-analysis of utility values for lung cancer. Med Decis Making. 2010;30(6):685-693. doi:10.1177/0272989X10369004 20448248

[16] Chen S. Study on Economic Burden and Quality of Life of Lung Cancer Patients. Anhui Medical University; 2016.

[17] Gareen IF, Duan F, Greco EM, . Impact of lung cancer screening results on participant health-related quality of life and state anxiety in the National Lung Screening Trial. Cancer. 2014;120(21):3401-3409. doi:10.1002/cncr.28833 25065710 PMC4205265

[18] He J, Chen W. China Cancer Registry Annual Report 2018. People’s Medical Publishing House; 2019.

[19] Yuan J, Sun Y, Wang K, . Cost effectiveness of lung cancer screening with low-dose CT in heavy smokers in China. Cancer Prev Res (Phila). Published online September 27, 2021. doi:10.1158/1940-6207.CAPR-21-0155 34580085

[20] Sun C, Zhang X, Guo S, . Determining cost-effectiveness of lung cancer screening in urban Chinese populations using a state-transition Markov model. BMJ Open. 2021;11(7):e046742. doi:10.1136/bmjopen-2020-046742 34210726 PMC8252866

[21] Chen WQ, Li N, Cao MM, . Preliminary analysis of cancer screening program in urban China from 2013 to 2017. China Cancer. 2020;29:1-6.

[22] Zhang M, Chunxiao W, Yangming G, . Survival analysis of patients with lung cancer in Shanghai. China Oncology. 2017;27(5):326-333.

[23] Tabulation on the 2010 Population Census of the People’s Republic of China. Department of Population and Employment Statistics, National Bureau of Statistics of China; 2010.

[24] Liu BQ, Peto R, Chen ZM, . Emerging tobacco hazards in China: 1. Retrospective proportional mortality study of one million deaths. BMJ. 1998;317(7170):1411-1422. doi:10.1136/bmj.317.7170.1411 9822393 PMC28719

[25] Wang DZ, Zhang H, Zhang Y, . A population-based case-control study on the relationship between smoking and lung cancer death. J Tuberculosis Lung Health. 2012;(2):6.

[26] Hong S, Mok Y, Jeon C, Jee SH, Samet JM. Tuberculosis, smoking and risk for lung cancer incidence and mortality. Int J Cancer. 2016;139(11):2447-2455. doi:10.1002/ijc.30384 27521774

[27] Sozzi G, Boeri M, Rossi M, . Clinical utility of a plasma-based miRNA signature classifier within computed tomography lung cancer screening: a correlative MILD trial study. J Clin Oncol. 2014;32(8):768-773. doi:10.1200/JCO.2013.50.4357 24419137 PMC4876348

[28] Ten Haaf K, van Rosmalen J, de Koning HJ. Lung cancer detectability by test, histology, stage, and gender: estimates from the NLST and the PLCO trials. Cancer Epidemiol Biomarkers Prev. 2015;24(1):154-161. doi:10.1158/1055-9965.EPI-14-0745 25312998 PMC4357842

[29] Shi JF, Wang L, Wu N, ; LuCCRES Group. Clinical characteristics and medical service utilization of lung cancer in China, 2005-2014: overall design and results from a multicenter retrospective epidemiologic survey. Lung Cancer. 2019;128(128):91-100. doi:10.1016/j.lungcan.2018.11.031 30642458

[30] Banerjee AK. Preinvasive lesions of the bronchus. *J Thorac Oncol*. 2009;4(4):545-551. 10.1097/JTO.0b013e31819667bd 19279508

[31] Neumann PJ, Cohen JT, Weinstein MC. Updating cost-effectiveness—the curious resilience of the $50,000-per-QALY threshold. N Engl J Med. 2014;371(9):796-797. doi:10.1056/NEJMp1405158 25162885

[32] Li N, Tan F, Chen W, ; National Lung Cancer Screening programme group. One-off low-dose CT for lung cancer screening in China: a multicentre, population-based, prospective cohort study. Lancet Respir Med. 2022;10(4):378-391. doi:10.1016/S2213-2600(21)00560-9 35276087

[33] Toumazis I, de Nijs K, Cao P, . Cost-effectiveness evaluation of the 2021 US Preventive Services Task Force recommendation for lung cancer screening. JAMA Oncol. 2021;7(12):1833-1842. doi:10.1001/jamaoncol.2021.4942 34673885 PMC8532037

[34] WTP threshold may influence ICERs for regulatory decisions. PharmacoEcon Outcomes News. 2018;800:33. doi:10.1007/s40274-018-4852-z

[35] Toumazis I, Erdogan SA, Bastani M, Leung A, Plevritis SK. A cost-effectiveness analysis of lung cancer screening with low-dose computed tomography and a diagnostic biomarker. *J Natl Cancer Inst Cancer Spectr*. Published online October 6, 2021. doi:10.1093/jncics/pkab081 PMC8564700 34738073

[36] Montani F, Marzi MJ, Dezi F, . miR-Test: a blood test for lung cancer early detection. J Natl Cancer Inst. 2015;107(6):djv063. doi:10.1093/jnci/djv063 25794889

[37] Zhao Z, Du L, Wang L, Wang Y, Yang Y, Dong H. Preferred lung cancer screening modalities in China: a discrete choice experiment. Cancers (Basel). 2021;13(23):6110. doi:10.3390/cancers13236110 34885217 PMC8656503

[38] Yongzhen Z, Qiusheng G, Wangfei C, . Analysis of screening results of early diagnosis and treatment of urban cancer in Shanxi Province from 2014 to 2018, China. Article in Chinese. Cancer. 2021;30:131-136.

[39] Guo LW, Zhang SK, Liu SZ, . [Compliance of lung cancer screening with low-dose computed tomography and influencing factors in urban area of Henan province]. Zhonghua Liu Xing Bing Xue Za Zhi. 2020;41(7):1076-1080. doi:10.3760/cma.j.cn112338-20190730-00564 32741174

[40] Le W, Xiaohua S, Meizhen Z. Preliminary analysis of screening results of early diagnosis and treatment of urban cancer in Zhejiang Province from 2013 to 2018, China. Cancer. 2020;29:904-909.

[41] McMahon PM, Kong CY, Bouzan C, . Cost-effectiveness of computed tomography screening for lung cancer in the United States. J Thorac Oncol. 2011;6(11):1841-1848. doi:10.1097/JTO.0b013e31822e59b3 21892105 PMC3202298

[42] Bach PB, Mirkin JN, Oliver TK, . Benefits and harms of CT screening for lung cancer: a systematic review. JAMA. 2012;307(22):2418-2429. doi:10.1001/jama.2012.5521 22610500 PMC3709596

[43] McCunney RJ, Li J. Radiation risks in lung cancer screening programs: a comparison with nuclear industry workers and atomic bomb survivors. Chest. 2014;145(3):618-624. doi:10.1378/chest.13-1420 24590022

[44] Goffin JR, Flanagan WM, Miller AB, . Cost-effectiveness of lung cancer screening in Canada. JAMA Oncol. 2015;1(6):807-813. doi:10.1001/jamaoncol.2015.2472 26226181

[45] Bethune R, Wu L, Goodridge D, . The clinical benefit and cost-effectiveness of adding a smoking cessation program to a simulated lung cancer screening program in Saskatchewan, Canada. Am J Respir Crit Care Med. 2017;195:AS179.

[46] Cao P, Jeon J, Levy DT, . Potential impact of cessation interventions at the point of lung cancer screening on lung cancer and overall mortality in the United States. J Thorac Oncol. 2020;15(7):1160-1169. doi:10.1016/j.jtho.2020.02.008 32160967 PMC7329583

[47] Cadham CJ, Cao P, Jayasekera J, ; CISNET-SCALE Collaboration. Cost-effectiveness of smoking cessation interventions in the lung cancer screening setting: a simulation study. J Natl Cancer Inst. 2021;113(8):1065-1073. doi:10.1093/jnci/djab002 33484569 PMC8502465

[48] Hofer F, Kauczor HU, Stargardt T. Cost-utility analysis of a potential lung cancer screening program for a high-risk population in Germany: a modelling approach. Lung Cancer. 2018;124:189-198. doi:10.1016/j.lungcan.2018.07.036 30268459

