# Supplementary material for: Cost-effectiveness of Low-Dose Computed Tomography With a Plasma-Based Biomarker for Lung Cancer Screening in China
Source: JAMA Netw Open. 2022 May 24;5(5):e2213634. doi: 10.1001/jamanetworkopen.2022.13634 (PMC9131747; doi:10.1001/jamanetworkopen.2022.13634)
Supplement: Supplement. — eTable 1. Sensitivity Analysis Range of Input Parameters for the Tornado Diagram eTable 2. Distributions for Input Parameters in Probabilistic Sensitivity Analysis eFigure. Scheme Diagram [file jamanetwopen-e2213634-s001.pdf]

Supplementary Online Content

Zhao Z, Wang Y, Wu W, Yang Y, Du L, Dong H. Cost-effectiveness of low-dose computed tomography with a plasma-based biomarker for lung cancer screening in China. *JAMA Netw Open*. 2022;5(5):e2213634. doi:10.1001/jamanetworkopen.2022.13634

eTable 1. Sensitivity Analysis Range of Input Parameters for the Tornado Diagram
eTable 2. Distributions for Input Parameters in Probabilistic Sensitivity Analysis
eFigure. Scheme Diagram

This supplementary material has been provided by the authors to give readers additional information about their work.

© 2022 Zhao Z et al. *JAMA Network Open*.

eTable 1. Sensitivity Analysis Range of Input Parameters for the Tornado Diagram

**Variables**	**Base-case value**	**Low entry**	**High entry**
Sensitivity of LDCT（%）	79	71	86
Specificity of LDCT（%）	81	73	89
Sensitivity of LDCT&MSC（%）	69	62	76
Specificity of LDCT&MSC（%）	96	86	100
Screening cost(LDCT, CNY)	245.86	122.93	368.79
Screening cost(MSC, CNY)	400	200	600
CPI rate	0.042	0.047	0.052
Discount rate	0.05	0	0.08

Abbreviations: LDCT, low-dose computed tomography; MSC, Micro-RNA signature classifier; CPI, Consumer Price Index.

eTable 2. Distributions for Input Parameters in Probabilistic Sensitivity Analysis

**Variables**	**Base-case value**	**Distribution**	**Mean, SD**
Lung cancer incidence rate in smokers(>20 pack-years)			
50-54	0.0016	Beta	(0.0016,0.0004)
55-59	0.0030	Beta	(0.0030,0.0003)
60-64	0.0047	Beta	(0.0047,0.0002)
65-69	0.0078	Beta	(0.0078,0.0002)
70-74	0.0104	Beta	(0.0104,0.0002)
Lung cancer incidence rate in smokers(>30 pack-years)		Beta	
50-54	0.0027	Beta	(0.0027,0.0001)

© 2022 Zhao Z et al. *JAMA Network Open*.

55-59	0.0045	Beta	(0.0045,0.0001)
60-64	0.0066	Beta	(0.0066,0.0002)
65-69	0.0091	Beta	(0.0091,0.0002)
70-74	0.0118	Beta	(0.0118,0.0002)
75-79	0.0130	Beta	(0.0130,0.0002)
Proportion of lung cancer by stage(LDCT only)			
CIS	0.0370	Beta	(0.0350,0.075)
I	0.6852	Beta	(0.7,0.1222)
II	0.0370	Beta	(0.035,0.075)
III	0.1852	Beta	(0.18,0.0366)
IV	0.0556	Beta	(0.05,0.0629)
Sensitivity of LDCT（%）	79	Beta	(0.79,0.0006)
Specificity of LDCT（%）	81	Beta	(0.81,0.0006)
Sensitivity of LDCT&MSC（%）	69	Beta	(0.69,0.0005)
Specificity of LDCT&MSC（%）	96	Beta	(0.96,0.0002)
Mortality of all-cause death for smokers			
50-54	0.0045	Beta	(0.0045,0.0005)
55-59	0.0065	Beta	(0.0065,0.0003)
60-64	0.0108	Beta	(0.0108,0.0002)
65-69	0.0188	Beta	(0.0188,0.0002)
70-74	0.0336	Beta	(0.0336,0.0001)
75-79	0.0540	Beta	(0.0540,0.0001)
Transition probabilities(1 year)			

© 2022 Zhao Z et al. *JAMA Network Open*.

Lung cancer stage CIS to lung cancer stage I	0.0980	Beta	(0.098,0.0005)
Lung cancer stage I to lung cancer stage II	0.3682	Beta	(0.3682,0.0008)
Lung cancer stage I to lung cancer stage III	0.0328	Beta	(0.0328,0.0005)
Lung cancer stage I to lung cancer stage IV	0.0745	Beta	(0.0745,0.0010)
Lung cancer stage II to lung cancer stage III	0.2260	Beta	(0.226,0.0010)
Lung cancer stage II to lung cancer stage IV	0.1510	Beta	(0.151,0.0011)
Lung cancer stage III to lung cancer stage IV	0.1455	Beta	(0.1455,0.0007)
Lung cancer stage CIS to death	0	Beta	-
Lung cancer stage I to death	0.1739	Beta	(0.1739,0.0008)
Lung cancer stage II to death	0.2842	Beta	(0.2842.0.0013)
Lung cancer stage III to death	0.4626	Beta	(0.4626,0.0053)
Lung cancer stage IV to death	0.5880	Beta	(0.5880,0.0042)
Utility			
CIS	0.87	Beta	(0.87,0.0022)
I	0.84	Beta	(0.84,0.0022)
II	0.84	Beta	(0.84,0.0022)
III	0.87	Beta	(0.87,0.0075)
IV	0.75	Beta	(0.75,0.0146)

© 2022 Zhao Z et al. *JAMA Network Open*.

Costs(CNY)			
Screening cost(LDCT)	245.86	Gamma	(245,63)
Screening cost(MSC)	400	Gamma	(400,6)
Pre-diagnosis cost	628.36	Gamma	(628,150)
Biopsy diagnosis cost	1232.44	Gamma	(1232,314)
Treatment cost			
CIS	47341.85	Gamma	(47342,7072)
I	53344.51	Gamma	(53345,16594)
II	83365.95	Gamma	(83365,8332)
III	90643.18	Gamma	(90643,5620)
IV	116471.34	Gamma	(116471,11501)

Abbreviations: LDCT, low-dose computed tomography; MSC, Micro-RNA signature classifier; CIS, carcinoma in situ; SD: standard deviation.

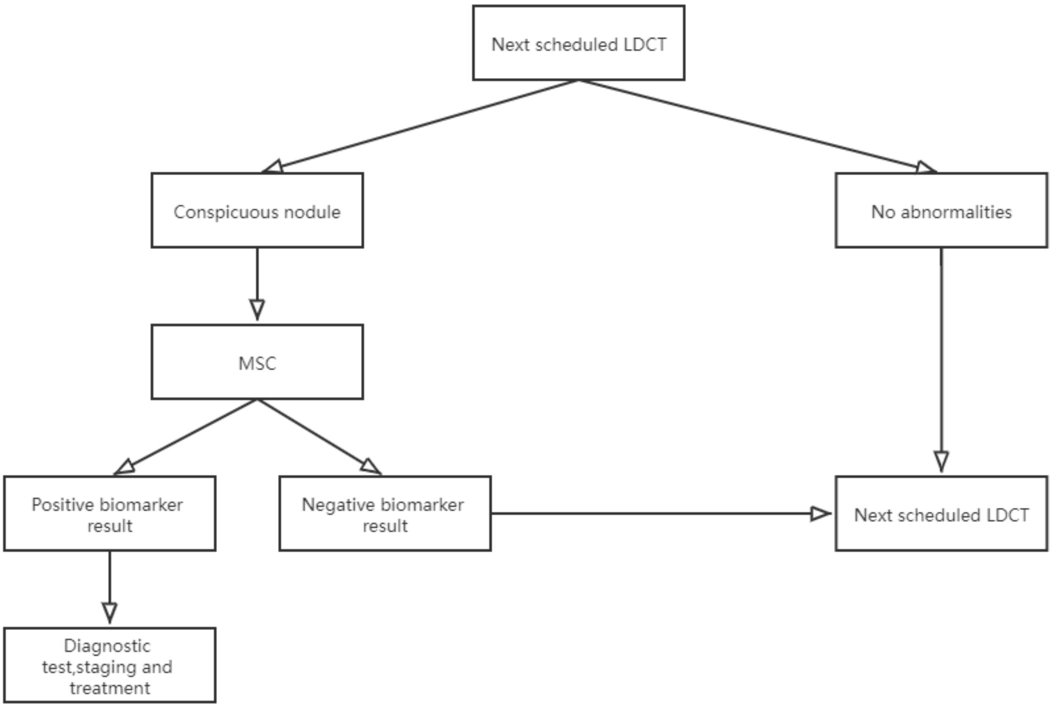

eFigure. Scheme Diagram

Abbreviations: LDCT, low-dose computed tomography; MSC, Micro-RNA signature classifier.

© 2022 Zhao Z et al. *JAMA Network Open*.
